# Hypoxia Affects Neprilysin Expression Through Caspase Activation and an APP Intracellular Domain-dependent Mechanism

**DOI:** 10.3389/fnins.2015.00426

**Published:** 2015-11-13

**Authors:** Caroline Kerridge, Daria I. Kozlova, Natalia N. Nalivaeva, Anthony J. Turner

**Affiliations:** ^1^Faculty of Biological Sciences, School of Molecular and Cellular Biology, University of LeedsLeeds, UK; ^2^Neuroscience, Eli Lilly and Company Limited, Lilly Research CentreSurrey, UK; ^3^I. M. Sechenov Institute of Evolutionary Physiology and Biochemistry, Russian Academy of SciencesSt. Petersburg, Russia

**Keywords:** neprilysin, hypoxia, caspases, AICD, APP

## Abstract

While gene mutations in the amyloid precursor protein (APP) and the presenilins lead to an accumulation of the amyloid β-peptide (Aβ) in the brain causing neurodegeneration and familial Alzheimer's disease (AD), over 95% of all AD cases are sporadic. Despite the pathologies being indistinguishable, relatively little is known about the mechanisms affecting generation of Aβ in the sporadic cases. Vascular disorders such as ischaemia and stroke are well established risk factors for the development of neurodegenerative diseases and systemic hypoxic episodes have been shown to increase Aβ production and accumulation. We have previously shown that hypoxia causes a significant decrease in the expression of the major Aβ-degrading enzyme neprilysin (NEP) which might deregulate Aβ clearance. Aβ itself is derived from the transmembrane APP along with several other biologically active metabolites including the C-terminal fragment (CTF) termed the APP intracellular domain (AICD), which regulates the expression of NEP and some other genes in neuronal cells. Here we show that in hypoxia there is a significantly increased expression of caspase-3, 8, and 9 in human neuroblastoma NB7 cells, which can degrade AICD. Using chromatin immunoprecipitation we have revealed that there was also a reduction of AICD bound to the NEP promoter region which underlies the decreased expression and activity of the enzyme under hypoxic conditions. Incubation of the cells with a caspase-3 inhibitor Z-DEVD-FMK could rescue the effect of hypoxia on NEP activity protecting the levels of AICD capable of binding the NEP promoter. These data suggest that activation of caspases might play an important role in regulation of NEP levels in the brain under pathological conditions such as hypoxia and ischaemia leading to a deficit of Aβ clearance and increasing the risk of development of AD.

## Introduction

Cerebral hypoxia is a condition in which the brain is deprived of oxygen and, as a result, cells elicit a wide range of adaptive responses and major metabolic alterations. Currently, vascular disorders such as ischaemia and stroke are considered as established risk factors for the development of neurodegenerative diseases, in particular of Alzheimer's disease (AD) (O'Brien and Markus, 2014). The pathology in familial AD is caused by overproduction and accumulation in the brain of abnormally high concentrations of the amyloid-β (Aβ) peptide and its oligomers causing synaptic loss and neuronal cell death (Hardy and Higgins, 1992; Walsh et al., 2002; Hardy, 2009). It is now becoming evident, however, that Aβ levels in the brain represent a dynamic equilibrium between its production from the amyloid precursor protein (APP) and removal by a cohort of amyloid clearance proteins which can be either enzymes (proteases) or binding/transport proteins. The group of enzymes capable of proteolytic degradation of Aβ currently embraces more than 20 members (for review, see Nalivaeva et al., 2012a, 2014). Several of the main amyloid-degrading enzymes (ADEs) are members of the neprilysin peptidase family: NEP, NEP2 and endothelin converting enzymes (ECE-1 and ECE-2) (Turner, 2003; Marr and Spencer, 2010; Nalivaeva et al., 2012a; Pacheco-Quinto and Eckman, 2013). Another metallopeptidase which plays an important role in Aβ metabolism is insulin-degrading enzyme (IDE) (Qiu and Folstein, 2006; Leissring and Turner, 2013). With aging and under pathological conditions expression levels and activity of these enzymes decline (Nalivaeva et al., 2004; Caccamo et al., 2005; Kochkina et al., 2015) leading to an amyloid clearance deficit, which is now considered to be one of the major factors of the sporadic form of AD (Selkoe, 2012; Pluta et al., 2013).

As we have shown earlier, hypoxia leads to reduced NEP levels and activity both in cellular models and *in vivo* (Nalivaeva et al., 2004, 2012b; Fisk et al., 2007). A prolonged exposure to a hypoxic environment has also been reported to increase Aβ levels significantly accelerating the hyperphosphorylation of tau and contributing to neuronal cell death (Jendroska et al., 1995; Li et al., 2009; Fang et al., 2010). Regulation of NEP expression is complex as the enzyme appears to have a constitutive regulatory pathway (D'Adamio et al., 1989; Li et al., 1995) as well as an epigenetically-regulated component (Pardossi-Piquard et al., 2005; Belyaev et al., 2009). The latter involves competitive binding of a transcription factor, namely the APP intracellular domain (AICD) produced in the β-secretase amyloidogenic pathway (Belyaev et al., 2010), to the NEP gene promoter leading to activation of mRNA synthesis while histone deacetylases inhibit this process. As the effects of hypoxia on NEP expression may represent an important pathological trigger in AD, the factors affecting NEP dysregulation under these conditions need to be better understood. AICD is an extremely labile peptide being a substrate of various intracellular peptidases including caspases (Bertrand et al., 2001) which might result in dysregulation of AICD-dependent NEP expression under various pathological conditions related to caspase activation. In particular, hypoxia was shown to be accompanied by increased levels of caspase expression and activity in the brain (Khurana et al., 2002). The aim of this study was to assess whether activation of caspases might be a factor leading to dysregulation of NEP gene expression and activity under hypoxic conditions. For this we have employed human neuroblastoma NB7 cells which possess high endogenous levels of NEP and, as has been demonstrated, are responsive to hypoxia (Fisk et al., 2007; Belyaev et al., 2009).

## Methods

### Cell culture and hypoxia treatment

The NB7 (SJ-N-CG) neuroblastoma cell line, which expresses high endogenous levels of NEP, was obtained from St Jude Children's Research Hospital (Memphis, USA, kind gift of Dr. Vincent J. Kidd). The NB7 cells were cultured in RPMI-1640 media supplemented with 10% (v/v) fetal bovine serum, 50 units/ml penicillin, 50 μg/ml streptomycin and 2 mM glutamine (all from Cambrex Bio Science Ltd., Wokingham, Berkshire, UK) at 37°C in 5% (v/v) CO_2_ and sub-cultured every 7 days. After reaching the confluent stage, cells were incubated in an O_2_/CO_2_ incubator (MC0-175M, Sanyo) for 24 h under 1% O_2_. The cells were collected 24 (or 48 h) later, washed twice with 10 ml PBS, scraped into 10 ml of PBS (pH 7.2), pelleted at 3000 g for 5 min and used for mRNA and protein content analysis as well as for the activity assays.

### Cell viability determination by trypan blue exclusion

Cells were washed twice in PBS, incubated in trypsin/EDTA for 5 min at 37°C and knocked from the surface of the flask prior to adding 5 ml of media then pelleting at 400 g for 5 min. Pellets were resuspended in 1 ml of media and a 1:1 dilution of cell suspension in 4% trypan blue was prepared. Twenty microlitres of cell suspension was loaded under a cover slip on a haemocytometer and allowed to fill the chambers by capillary action. The number of total and viable cells was determined by viewing under a light microscope at 40X magnification. Non-viable cells take up the trypan blue and appear dark (Strober, 2001).

### Gene expression analysis

Cell RNA was prepared using the RNeasy extraction kit (Qiagen, Crawley, UK) according to the manufacturer's protocol. RNA was treated with DNase I (Invitrogen, Paisley, UK) and cDNA was prepared using the iScript cDNA kit (BioRad, UK). cDNA was amplified using conventional PCR or real-time PCR as in Zuccato et al. (2007). DNA amplified by conventional PCR was analyzed in 2% agarose gels containing ethidium bromide (1 μg/ml) and visualized on a Molecular Imager Gel Doc XR System with Quantity One 4.6.1 programme (BioRad). Image densitometry was performed using Aida Array Analyzer 4.15 software. Real-time PCR was performed in an iCycler Thermal Cycler with Multicolour PCR detection system, (Biorad, Hercules, CA) using SYBR Green (BioRad) incorporation and expression reported relative to actin mRNA. Primer sequences used were as follows:

NEP F- CCTGGAGATTCATAATGGATCTTGT

R- AAAGGGCCTTGCGGAAAG

Caspase-1 F- TGCTTTCTGCTCTTCAACACC

R-CACAAGACCAGGCATATTCTTTC

Caspase-3 F-GAGGCCGACTTCTTGTATGC

R-AATTCTGTTGCCACCTTTCG

Caspase-8 F-AGAGCCTGAGGGAAAGATGTC

R-TCACATCATAGTTCACGCCAGT

Caspase-9 F-CGTGGTGGTCATCCTCTCTC

R-GAGCATCCATCTGTGCCATA

U6 F- CTCGCTTCGGCAGCACA

R- AACGCTTCACGAATTTGCGT

Actin F- CGCAGCAGTCAGGGACATTT

R- TTCACATACAGCTTGGGAAGC

### Preparation of cell lysates

Cells were washed twice with phosphate-buffered saline (PBS: 1.5 mM KH_2_PO4, 2.7 mM Na_2_HPO4, 150 mM NaCl, pH 7.4), harvested and pelleted by centrifugation. For detection of proteins from cell lysates, cells were lysed in RIPA buffer [10 mM Tris/HCl pH 8.0, 150 mM NaCl, 1% (v/v) Nonidet P-40, 0.5% (w/v) sodium deoxycholate, 5 mM EDTA, 1X complete inhibitor mix (Roche diagnostics)] on ice for 20 min. Lysates were homogenized through 22 G needles 10 times then clarified by centrifugation at 2800 g for 10 min. Protein concentration of lysates was determined using the bicinchoninic acid (BCA) assay (Sigma Aldrich).

### Electrophoresis and western blotting

Samples (30 μg or, for AICD detection, 50 μg, protein) were resolved on 10% polyacrylamide gels or, for AICD detection, 10–20% tricine gels (Invitrogen, Paisley, UK), and transferred onto Hybond-P poly(vinylidene) difluoride (PVDF) membranes (Amersham Life Sciences, Buckinghamshire, UK). The membranes were blocked overnight at 4°C in Tris-buffered saline (TBS: 50 mM Tris, 150 mM NaCl) containing 0.1% (v/v) Tween-20 (TBST) and 5% (w/v) skimmed milk powder. Membranes were incubated with the primary antibodies for 3 h. Primary antibodies used for blotting included: NEP (1:100, mouse monoclonal, NCL-CD10-270, Novocastra, Newcastle, UK or rabbit anti-rat polyclonal antibody, US Biological); GLUT-1 (1:500, rabbit polyclonal, from Prof. S. Baldwin, University of Leeds); anti- β-actin (1:500, rabbit polyclonal, Sigma-Aldrich Co., Poole, Dorset, UK); anti-APP C-Terminal fragment A8717 and anti-actin (both Sigma-Aldrich) used at 1:1000 and 1:10,000, respectively. Membranes were washed in TBST for 1 h before incubation with peroxidase-conjugated rabbit anti-mouse or donkey anti-rabbit secondary antibodies (GE Healthcare, Bucks, UK) at a dilution of 1:4000. Membranes were washed further before detection of proteins using the enhanced chemiluminescence method. Western blots were quantified using AIDA image analyzer v.4.22 (Straubenhardt, Germany).

### Neprilysin activity assay

NEP activity was measured in a two-step coupled fluorescence assay. NEP cleaves between Ala-Phe of the fluorogenic substrate succinoyl-Ala-Ala-Phe-7-amido-4-methylcoumarin (Mumford et al., 1980) and, in the presence of leucine aminopeptidase, releases the fluorophore (AMC) from Phe and prevents quenching allowing fluorescence to be detected. Protein samples (1 μg) were loaded into opaque 96-well plates in 100 mM Tris-HCl (pH 7.4) with or without 20 μM of the NEP inhibitor thiorphan and incubated at 37°C for 20 min. The substrate Suc-AAF-AMC (50 μM) and Leu-aminopeptidase (40 munits/ml) were added to each well (100 μl total volume/well) and fluorescence was observed on a FLUOstar Omega multidetection microplate reader (excitation = 355 nm, emission = 460 nm) at suitably frequent time points. NEP activity was determined as the difference between fluorescence measurements in the absence and presence of 20 μM thiorphan.

### Caspase 3/7 activity assay

Caspase-3 and caspase-7 activities were determined using a luminescent Caspase-Glo® 3/7 activity assay (Promega, Southampton, UK) as per manufacturers' instruction. Briefly, approximately 20,000 cells were added to each well of a 96 well, white-walled plate. At room temperature, the Caspase-Glo reagent was made up by mixing the Caspase-Glo substrate with the Caspase-Glo buffer and 100 μl was added to each well. Contents were mixed by shaking for 1 min then incubated at 37°C for 1 h. Luminescence was measured on a MicroLumat Plus LB 96V (Berthold Technologies).

### Chromatin immunoprecipitation analysis

ChIP was performed as described previously (Zuccato et al., 2007; Belyaev et al., 2009). Cells were fixed, extracts sonicated and, following incubation with protein-G-Sepharose, primary antibodies were applied overnight. After de-crosslinking and DNA extraction, analysis was performed by real time PCR. Data are represented as the fold enrichment of DNA pulled down with the specific antibody over that immunoprecipitated with IgG. Antibodies used were anti-AICD (Covance, Harrogate) and anti-IgG (mouse) (negative control) (Abcam). Promoter primer sequences used were as follows:

NEP F- GGTGCGGGTCGGAGGGATGC

R- CTCCCAGCGCCCTGGGCGCTCG

### Statistics

All results are given as mean ± SEM from at least three experiments. Results were compared using unpaired two-tailed Student's *t*-test with a threshold of *p* < 0.05.

## Results

### Cell viability and GLUT1 expression in NB7 cells after hypoxia

**Figure 1:**
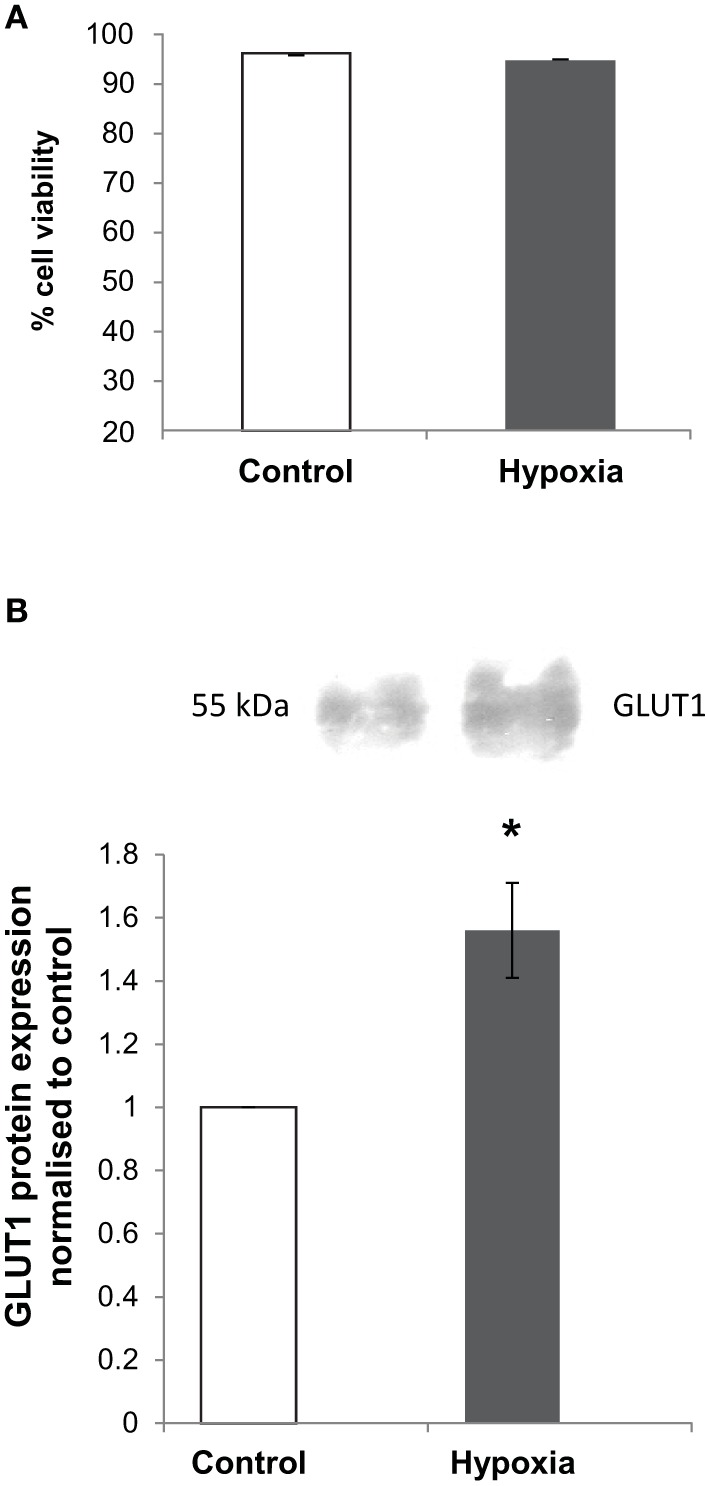
**The effects of hypoxia on cell viability and GLUT1 protein levels in NB7 cells. (A)** A cell viability assay carried out in NB7 cells subjected to 24 h normoxia or hypoxia (1% O_2_). **(B)** Representative Western blot and calculation of GLUT1 levels in whole cell lysates of NB7 cells subjected to 24 h normoxic or hypoxic (1% O_2_) conditions. Bars represent mean ± SEM, ^*^p < 0.05 (*n* = 3).

Hypoxia elicits a wide range of cellular adaptations and the severity and duration of a hypoxic episode can significantly affect hypoxia-induced apoptosis (Banasiak and Haddad, 1998). In order to show that the NB7 cells were responding to hypoxic conditions and were still viable, a cell viability assay was carried out as well as a Western blot analysis of the established hypoxia marker, GLUT1. Acting through the hypoxia- inducible factor-1, GLUT-1 expression is enhanced in hypoxia, stimulating glucose transport and maintaining cell homeostasis (Ouiddir et al., 1999; Zhang et al., 1999). After incubation at 1% O_2_ for 24 h, the viability of NB7 cells was not significantly different from those incubated under normal conditions (Figure 1A). In both conditions cell viability was above 90%. Although GLUT1 protein content was detected at high levels in both normoxia and hypoxia incubated cells, 24 h of hypoxia increased GLUT-1 protein levels significantly suggesting that the cells were responding to hypoxic insult by changing the level of expression of hypoxia-related genes (Figure 1B).

### The effects of hypoxia on NEP mRNA expression, protein level, and activity

**Figure 2:**
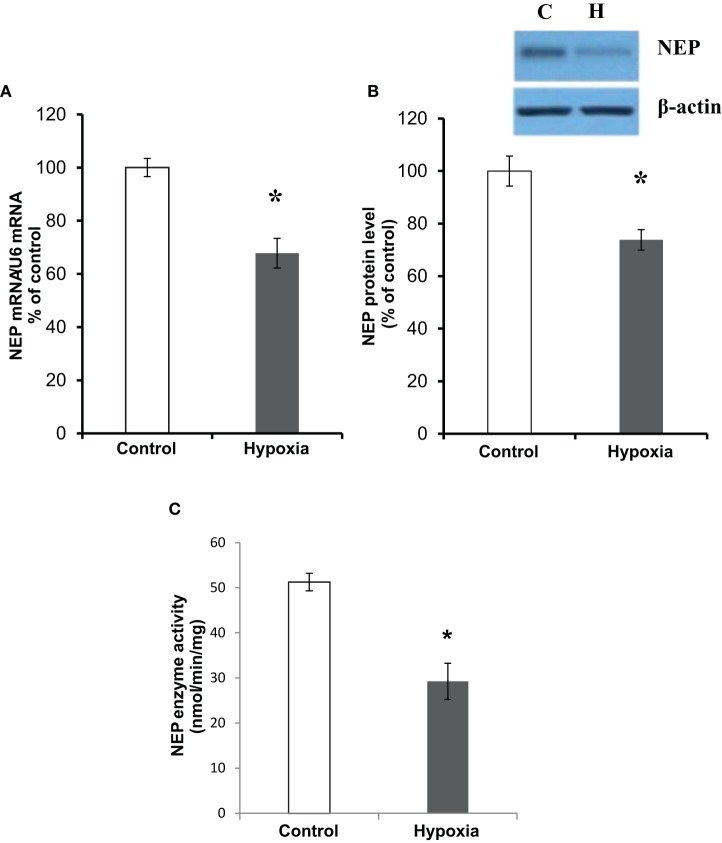
**The effects of hypoxia on NEP expression and activity. (A)** NEP mRNA expression levels normalized to U6; **(B)** NEP protein levels showing a representative blot with CD10 antibody against human NEP and β-actin as loading control; **(C)** NEP enzyme activity (nmol/min/mg) in NB7 cells subjected to 24 h normoxia or 24 h hypoxia (1% O_2_). Bars represent mean ± SEM, ^*^p < 0.05 (*n* = 5).

Assessing the effect of hypoxia on NEP expression in NB7 cells we have confirmed that both NEP mRNA and protein levels in the cells subjected to hypoxia for 24 h were reduced by approximately 30% compared to the control cells (Figures 2A,B). Similarly, a fluorogenic NEP enzyme activity assay has demonstrated that NEP activity was reduced in hypoxia by approximately 40% (Figure 2C).

### The effects of hypoxia on mRNA expression and activity of members of the caspase family

**Figure 3:**
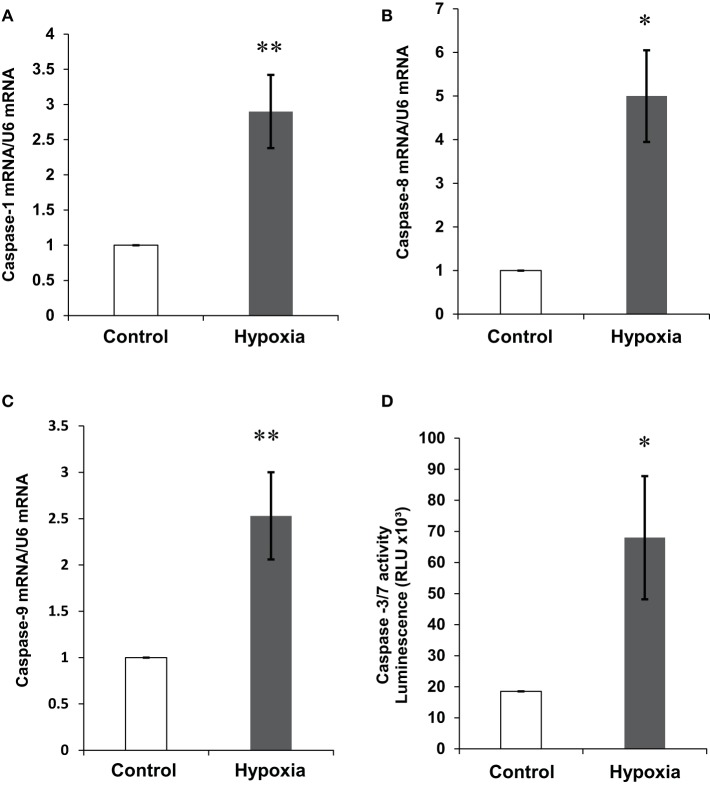
**The effect of hypoxia on caspase-1,-8, and -9 mRNA expression and caspase -3/-7 activity levels**. Expression levels of **(A)** caspase-1, **(B)** caspase-8, **(C)** caspase-9 normalized to U6 in NB7 cells subjected to 24 h normoxia or 24 h hypoxia (1% O_2_). **(D)** Caspase-3/-7 activity levels in NB7 cells subjected to 24 h normoxia or 24 h hypoxia (1% O_2_). Bars represent mean ± SEM, ^*^p < 0.05, ^**^p < 0.01.

Caspases are a family of cysteine proteases that are crucial mediators of apoptosis. Hypoxic conditions are known to induce cellular apoptosis and the family of caspases are participants in the hypoxia-induced apoptotic cascade (Khurana et al., 2002; Li et al., 2005). In order to investigate the effect of hypoxia on the expression of caspases in our cellular model, mRNA levels for caspases-1, -8, and -9 were determined by real-time PCR and a caspase-3/-7 activity assay was also carried out. A three to five-fold increase in mRNA was detected for caspase-1 and -9 (Figures 3A,C). In accordance with other studies (Finlay et al., 2010), caspase-8 expression was extremely low in the NB7 cells. However, despite endogenously low levels, hypoxia also increased its mRNA expression (Figure 3B). Since caspase-3 and caspase-7 exhibit similar substrate specificity, the analysis of their activity was combined. Total caspase -3/-7 activity was increased by approximately four-fold in the NB7 cells incubated in hypoxic conditions compared with control (Figure 3D).

### The effect of hypoxia on AICD enrichment of the NEP promoter

**Figure 4:**
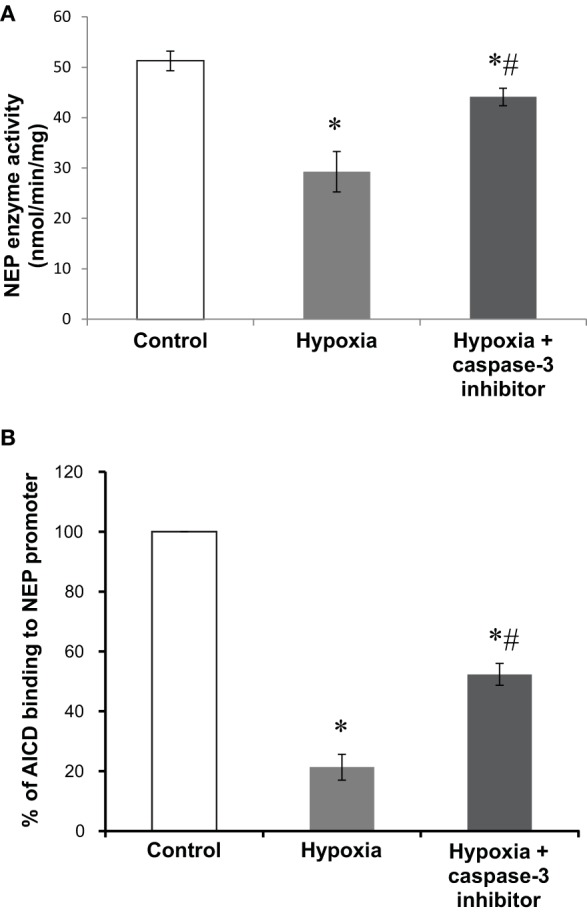
**The effect of a caspase-3 inhibitor on NEP activity and AICD binding to the NEP promoter in hypoxia. (A)** NEP enzyme activity (nmol/min/mg) and **(B)** enrichment of AICD on the NEP promoter analyzed by ChIP in NB7 cells subjected to 24 h normoxia or 24 h hypoxia (1% O_2_) with or without the caspase-3 inhibitor, Z-DEVD-FMK (100 μM, 24 h). Bars represent *n* of 3 ± SEM, ^*^p < 0.05 compared to control, ^#^*p* < 0.05 compared to hypoxia.

The APP C-terminal fragments (CTFs) have been identified as a target for caspase cleavage due to the presence of a caspase-specific cleavage site at aspartate residue 664 (for APP_695_ isoform) in the APP molecule (Weidemann et al., 1999). As caspases appear to be significantly elevated in hypoxia, ChIP analysis was used to determine whether the reduction in NEP may be due to reduced AICD binding to the *NEP* promoter region. After 24 h hypoxic incubation, AICD enrichment on the *NEP* promoter region was found to be significantly reduced by approximately five-fold (Figure 4B).

### The effects of caspase inhibition in hypoxia on NEP activity and AICD enrichment on the NEP promoter

Caspase-3 has been identified as the predominant caspase involved in the cleavage of the APP-CTF, including AICD (Gervais et al., 1999), therefore in order to investigate whether caspase-3 is involved in the reduction of AICD-induced NEP expression, cells were subjected to hypoxia with or without the caspase-3 inhibitor, Z-DEVD-FMK. As previously shown, NEP activity was significantly reduced in hypoxia compared to control, but cell treatment with the caspase-3 inhibitor restored NEP activity to approximately 90% of the control level (Figure 4A). Similarly, ChIP analysis has revealed that caspase inhibitor treatment to a significant extent protected the level of AICD binding to the NEP gene promoter (Figure 4B). This testifies to the role of caspases, and especially of caspase-3, in reduction of NEP expression in neuronal cells subjected to hypoxic conditions.

## Discussion

Hypoxia is an established factor for AD and brain deprivation of oxygen has been associated with an increase in Aβ levels and plaque deposition (Jendroska et al., 1995; Zhang and Le, 2010). The leading factor in Aβ accumulation might be deficit of its major degrading peptidase, NEP. Indeed, dysregulation of NEP after exposure to hypoxia has been reported in many areas of the body including the lungs (Carpenter and Stenmark, 2001; Wick et al., 2011), carotid body (Kumar et al., 1990), eye (Hara et al., 2006), and brain (Nalivaeva et al., 2004, 2012b; Oh-hashi et al., 2005). The data of the present study report significantly reduced NEP expression and activity in the human neuroblastoma NB7 cell line subjected to hypoxia, which are consistent with other reports.

Oxygen homeostasis is regulated by the hypoxia inducible factor (HIF)-1α (for review see Semenza, 2006). HIF-1α binds specifically to the hypoxia-responsive element (HRE) in gene promoter regions and in hypoxic conditions which can significantly alter cellular gene expression. The effects of hypoxia on APP processing have been widely studied and it is now well established that hypoxia increases the amyloidogenic pathway by facilitating the up-regulation of expression of β-secretase (BACE1), as well as components of the γ-secretase complex resulting in increased production both of Aβ_40_ and Aβ_42_ (Sun et al., 2006; Wang et al., 2006; Zhang et al., 2007; Li et al., 2009). Sequence analysis, mutagenesis and gel shift studies have revealed the binding of HIF-1α to both the BACE1 and APH-1A promoter regions and Notch processing has also been increased after hypoxic incubation (Wang et al., 2006; Zhang et al., 2007). Hypoxia has also been shown to decrease levels of metalloproteinases ADAM10 and ADAM17 expression and consequently reduce APP processing through the non-amyloidogenic, α-secretase pathway (Webster et al., 2004; Rybnikova et al., 2012). A reduction in mature ADAM10 and a concomitant increase in immature ADAM10 has also been reported to alter the regulation of α-secretase APP processing in hypoxia (Auerbach and Vinters, 2006).

The data in this paper provide evidence for an alternative mechanism by which hypoxia may increase Aβ levels. The hypoxia-induced activation of caspases may cause an increased cleavage of the APP-CTF and thus reduce AICD levels. This then results in the down-regulation of the AICD-mediated gene NEP and reduction of enzyme expression and activity which inevitably will lead to increase of Aβ content. It has been proven both in cell and animal models that NEP deficit is one of the factors leading to increased Aβ production and load in the brain (Iwata et al., 2000; Hanson et al., 2011) and up-regulation of NEP, on the contrary, leads to reduction of Aβ levels (Mohajeri et al., 2004; Kerridge et al., 2014). We have also shown that caspase-3 inhibition using the potent DEVD caspase inhibitor can protect NEP activity in the hypoxic conditions. Similar findings have been reported in dying motor-neurons deprived of trophic support. This type of cellular stress was shown to cause an increase in the expression of APP and the cell death protease caspase-3, as well as a concomitant increase in neurotoxic Aβ (Barnes et al., 1998). Although AICD and NEP levels were not investigated, treatment with a caspase-3 inhibitor significantly reduced the Aβ peptide content in these types of cells. Apoptotic stimuli activating the caspase-3 protease family have also been reported to cleave both PS1 and PS2 (Kim et al., 1997). As such, an alternative mechanism could involve an altered γ-secretase cleavage of APP thus generating APP-CTF incapable of translocating to the nucleus and/or regulating the transcription of NEP.

It was demonstrated that hypoxia affects NEP gene expression via histone modifications on its promoter region (Wang et al., 2011) which can affect accessibility of AICD to its binding site. As we have shown here, AICD binding to the NEP gene promoter is indeed reduced under hypoxic conditions but this decrease can be prevented by a caspase inhibitor. Because inhibition of caspase activity does not restore completely the level of AICD binding to the NEP promoter observed in the control cells, it is reasonable to suggest that either there are other peptidases active under hypoxic conditions that degrade AICD, or changes in the NEP promoter structure due to histone deacetylation are still interfering with its activation by AICD. As we have shown previously (Belyaev et al., 2009; Nalivaeva et al., 2012b), inhibition of histone deacetylases by valproic acid leads to significant but not complete restoration of NEP gene expression and NEP enzyme activity after hypoxia which confirms the role of histone modifications in NEP regulation under reduced oxygen supply.

AICD was also shown to regulate HIF-1α expression (Kaufmann et al., 2013) and, therefore, decreased levels of this transcription factor under hypoxic conditions might result in a modified response of cells to hypoxic insults. As we have established, the Aβ-clearing protein TTR is also AICD-regulated (Kerridge et al., 2014) and is shown to be responsive to hypoxia (Ahmad et al., 2014). Alterations of the levels of this protein under hypoxic conditions may also affect amyloid metabolism leading to dysbalance of its production and clearance.

There is an increasing body of evidence that AICD levels can be regulated by various pharmacological agents (Eisele et al., 2007; Bauer et al., 2011; Kerridge et al., 2014) which opens an avenue for designing a therapeutic strategy for regulating important AICD-dependent genes. Because AICD-regulated genes have different roles in amyloid metabolism and are cell- and tissue-specific (for review see Beckett et al., 2012; Pardossi-Piquard and Checler, 2012) understanding the effect of various physiological conditions on its ability to regulate expression of the major amyloid-degrading enzyme is of great importance. This work extends further our knowledge about the role of hypoxia in NEP regulation via AICD and shows that caspase inhibitors can be potentially beneficial for protecting the brain against harmful effects of low oxygen supply.

### Conflict of interest statement

The authors declare that the research was conducted in the absence of any commercial or financial relationships that could be construed as a potential conflict of interest.
